# Chromosomal-scale genome assembly and annotation of the land slug (*Meghimatium bilineatum*)

**DOI:** 10.1038/s41597-023-02893-7

**Published:** 2024-01-05

**Authors:** Shaolei Sun, Xiaolu Han, Zhiqiang Han, Qi Liu

**Affiliations:** 1 Fishery College, Zhejiang Ocean University, Zhoushan, Zhejiang 316022 China; 2 Wuhan Onemore-tech Co., Ltd, Wuhan, Hubei 430076 China

**Keywords:** Genome, Phylogenetics

## Abstract

*Meghimatium bilineatum* is a notorious pest land slug used as a medicinal resource to treat ailments in China. Although this no-model species is unique in terms of their ecological security and medicinal value, the genome resource of this slug is lacking to date. Here, we used the Illumina, PacBio, and Hi-C sequencing techniques to construct a chromosomal-level genome of *M. bilineatum*. With the Hi-C correction, the sequencing data from PacBio system generated a 1.61 Gb assembly with a scaffold N50 of 68.08 Mb, and anchored to 25 chromosomes. The estimated assembly completeness at 91.70% was obtained using BUSCO methods. The repeat sequence content in the assembled genome was 72.51%, which mainly comprises 34.08% long interspersed elements. We further identified 18631 protein-coding genes in the assembled genome. A total of 15569 protein-coding genes were successfully annotated. This genome assembly becomes an important resource for studying the ecological adaptation and potential medicinal molecular basis of *M. bilineatum*.

## Background & Summary

The *Meghimatium bilineatum* (*syn. Philomycus bilineatus* Benson, 1842) is a member of the Philomycidae family and is a notorious quarantine pest land slug that can cause enormous damage to commercial crops, horticultural crops, grasslands, and forests in East Asia^1–5^. It has a strong ecological adaptation to terrestrial environments and has been widely distributed in various regions of China^6^. It does not only feed on stems, leaves, fruits, or juices of plants causing direct economic losses but also secretes mucus and excretes feces contaminating fruits and vegetables. This contamination results in a reduction in the market value of products and transmits diseases. Thus, it poses great harm to local agricultural productivity and ecological security, resulting in substantial economic and ecosystem losses^7^. However, from another perspective, *M. bilineatum* also exhibits medicinal properties. For example, its crude extracts are used in the treatment of bacterial-induced infectious diseases, the polysaccharides in slug cell are used as natural antioxidants to prevent cancer, and the antimicrobial peptide derived from the slug is utilized to combat skin infections caused by *Candida albicans*^8–10^. At present, some researchers have carried out in-depth studies on the pharmacological effects of slug extract, indicating that slugs can be used as a valuable medicinal resource with development and application value^9,10^. Thus, the study of slug species is very meaningful.

In addition to its ecological threat and medicinal value, *M. bilineatum*, as a member of 30000 described terrestrial gastropod mollusks with shell-less, has completed the transition from aquatic to terrestrial. Similar to other slug species, they have developed many various robust features, including a pulmonate for breathing air, a sophisticated neural-immune system, and the ability to produce mucus to adapt to the terrestrial environments^11–13^. However, compared with land snails, land slugs display unique life strategy for terrestrial environments, such as defense by secreting mucus including specific chemical compounds and better mobility under predation, because they have no protective shell^1,14^. Furthermore, shell-less land slugs do not expend energy ingesting large amounts of calcium, enabling them to grow faster. Although land slugs have strong adaptation mechanism, their evolutionary history remains unclear. In recent years, molecular phylogenetics analysis of land slugs of the genus *Meghimatium* based on the mitogenome and nuclear loci has offered new perspectives into the taxonomic revisions and evolution of these species^15–17^. However, these studies cannot fully explain the molecular mechanism of wide ecological adaptation information and the potential genetic basis of medicinal resource traits of this slug. Furthermore, the Philomycidae slug genomics have yet to be published. Therefore, assembling a genome of this slug species should be urgently assembled.

The study of genomes in certain terrestrial mollusks, has shown advancements, including the release of genomic data for two land snails, *Achatina fulica* and *Pomacea canaliculata*. However, thorough investigations into the evolutionary mechanisms associated with terrestrial adaptation remain scant^18,19^. Recently, one genome study of *Achatina immaculata*, namely giant African snail has verified that some genes related to respiratory system, dormancy system, and immune system have undergone great expansion to adapt to the terrestrial environments^20^. However, to date, high-quality genomic resources for land slugs are rarely reported. The land slugs and snails, as terrestrial gastropod mollusks with or without shell protection, have different biological processes related to their terrestrial lifestyle. Hence, assembling a genome of the land slug species would facilitate intensive study of this species’ adaptive evolution.

Herein, we assembled the genome of *M. bilineatum* by uniting the sequencing techniques of Illumina, PacBio, and Hi-C. Three methods, including *ab initio* gene prediction, homolog and RNA-Seq-based prediction, were used to perform genomic annotation. In addition, the comparative genomics analysis of *M. bilineatum* and 11 other distantly related species were performed. This study offers insights for the effective management and utilization of slug populations and provides valuable genome information into the evolutionary history and genetic mechanisms of this important gastropod group.

## Methods

### Land slug collecting and sequencing

Adult land slugs *M. bilineatum* were collected from a wild area in Zhoushan, Zhejiang, China (122.212 E, 29.979 N). Total DNA was extracted from whole body of the land slug *M. bilineatum* using the SDS-based extraction method. Then, the DNA samples were purified using QIAGEN^®^ Genomic kit (QIAGEN, Germany) for genome sequencing. First, Illumina short-read library with insert sizes of 300–350 bp was generated, and was sequenced using the Illumina Novaseq. 6000 platform. Second, PacBio HiFi-read library with insert sizes of 10–40 kb was generated using SMRTbell Express Template Prep Kit 2.0 (Pacific Biosciences, USA) and sequenced using the PacBio Sequel II platform. Finally, Hi-C short-read library was generated using the purified DNA from the whole body of *M. bilineatum* according to the previously performed protocol by Belton *et al*. with given adjustments; it was sequenced using the Illumina Novaseq. 6000 platform^21^. A total of 250.12 Gb of clean Illumina short-reads, 71.33 Gb HiFi CCS reads and 140.69 Gb clean Hi-C reads were obtained (Table 1).

**Table 1:** Statistics of sequencing read data.

Libraries	Clean reads number	Clean data (Gb)	Read length (bp)	GC content (%)
Illumina reads	1,673,583,920	250.12	149	37.15
PacBio reads	3,827,020	71.33	18,637.99	37.06
Hi-C reads	945,397,772	141.81	150	38.32
RNA-seq	43,574,128	6.54	150	32.77
Total	2,666,382,840	469.80	–	–

Total RNA was isolated from whole body of the land slug using TRIzol reagent (Invitrogen, MA, USA) for transcriptome sequencing. The RNA-seq library was generated using NEBNext^®^ Ultra^™^ RNA Library Prep Kit (NEB, USA) and sequenced using the Illumina Novaseq. 6000 platform. The RNA-seq reads were used for genome annotation. A total of 21.79 Gb of clean data was obtained (Table 1).

### Genome size estimation

**Fig. 1:**
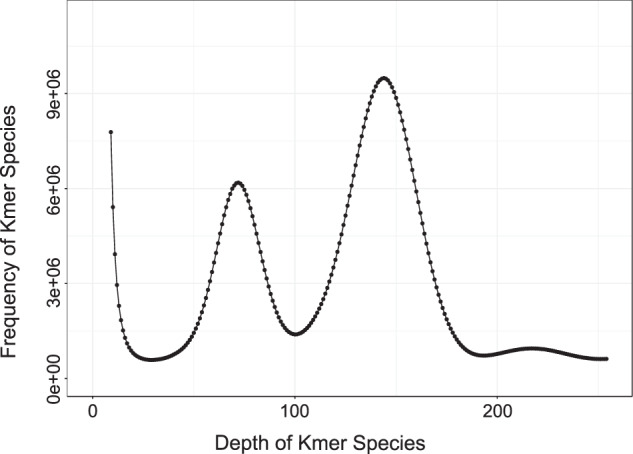
K-mer (17-mer) distribution and estimation of genome size of *M. bilineatum*.

Based on 250.12 Gb clean Illumina short-reads, the genome size, heterozygosity and repetitive sequence content was determined using the k-mer analysis with GCE (1.0.0) following the default parameter^22^. A total of 223,346,880,670 k-mers with a depth of 144 was obtained (Fig. 1). In addition, the genome size of *M. bilineatum* was approximately 1.5 Gb, with a heterozygosity of 1.05% and proportion of repeat sequences at 43.69%.

**Fig. 2:**
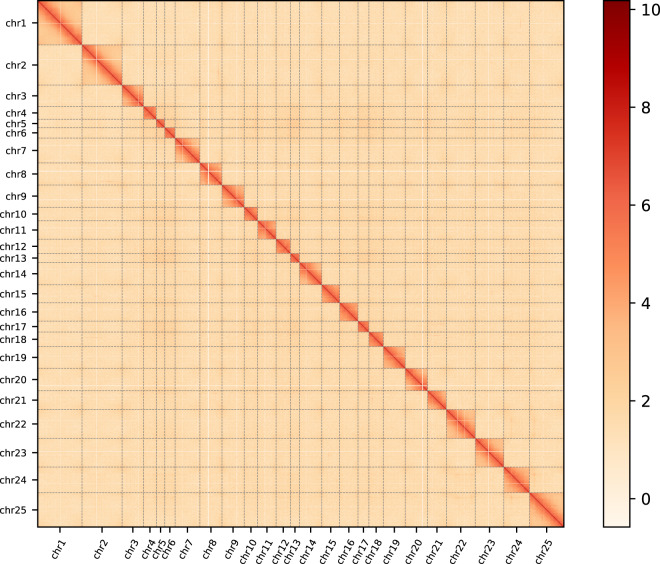
Chromosomal Hi-C heatmap of the *M. bilineatum* genome assembly.

### Chromosomal-level genome assembly

In the initial genome assembly, HiFiasm (v0.16.0) method was used for *ab initio* to assemble the genome using the HiFi reads from PacBio^23^. This preliminary assembly yielded a genome size of 1.80 Gb (Table 2). Subsequently, the redundant sequences were filtered out using Purge_Haplotigs (v1.0.4) software with the parameter of cutoff ‘-a 70 -j 80 -d 200’^24^. Based on PacBio sequencing data, a 1.63 Gb contig-level genome assembly of *M. bilineatum* was obtained, and 2526 contigs displayed contig N50 and N90 sizes of 1.37 and 320.449 Mb, respectively (Table 2). The chromosome-level assembly of *M. bilineatum* was conducted using Hi-C technology. Initially, Bowtie2 (v2.3.4.3) following the default parameters was used to match the 140.69 Gb clean Hi-C reads to the contig-level genome to obtain unique mapped paired-end reads^25^. A total of 185.36 million paired-end reads were uniquely mapped (Table S1), of which 88.02% represented valid pairs (Table S2). Subsequently, contigs were assembled into the chromosome-level scaffolds using the 3D-DNA processes (v180922) (parameters: -r 0) with all valid pairs, and the JuiceBox (v1.11.08) was used to correct the errors in the genome assembly^26,27^. We anchored and obtained 25 pseudo-chromosomes with seven unanchored scaffolds. The 25 pseudo-chromosomes covering ~99.95% of the final genome with size ranging from 25.66 Mb to 135.71 Mb (Fig. 2; Table 3). Ultimately, we obtained a 1.61 Gb chromosomal-level genome assembly of *M. bilineatum* with contig N50 size and scaffold N50 size of 1.36 Mb and 68.08 Mb, respectively. Genome assembly results showed that the genome size of *M. bilineatum* is similar to that of the Spanish slug *Arion vulgaris* (1.54 Gb) in the previous study^28^.

**Table 2:** Number and length statistics for the *M. bilineatum* genome assembly.

Mode	Total length (Gb)	Total number	N50 (Mb)	N90 (Mb)	GC content (%)
Hifiasm	1.80	3782	1.21	226.655	37.08
Hifiasm + Purge_Halotigs	1.63	2526	1.37	320.449	37.08

**Table 3:** Chromosome sizes and assignment for Hi-C scaffolds.

Pseudomolecule	Conting number	Length (Mb)
chr1	330	135.71
chr2	266	122.74
chr3	85	65.87
chr4	42	38.99
chr5	14	25.66
chr6	13	31.77
chr7	97	76.04
chr8	250	67.65
chr9	81	68.05
chr10	38	41.46
chr11	61	56.40
chr12	42	43.76
chr13	11	27.87
chr14	97	68.08
chr15	76	54.98
chr16	85	56.09
chr17	25	33.46
chr18	49	44.30
chr19	100	66.95
chr20	103	68.48
chr21	90	57.31
chr22	91	88.69
chr23	171	87.48
chr24	91	78.64
chr25	213	105.18
Total anchored	2521	1611.61
Unanchored	7	0.77

### Repeat-content identification and classification

Repetitive sequences, including tandem repeats and interspersed repeats, in *M. bilineatum* genome were determined using the *de novo* prediction and homolog-based methods. Based on homology comparison, RepeatMasker (open-4.0.9) (parameters: default) and RepeatProteinMask (parameters: default) software were utilized to find the interspersed repeats against the RepBase database (http://www.girinst.org/repbase)^29^. On the basis of *de novo* prediction, TRF (v4.09) software (parameters: default) was used to identify the tandem repeats^30^. In addition, a repetitive sequence library was constructed using the RepeatModeler (open-1.0.11) with default parameters and LTR-FINDER_parallel (v1.0.7) with default parameters^31,32^. Then, the RepeatMasker (open-4.0.9) with default parameters was used to identify the repeat element against this repeat library^31^. After combining the results from *de novo* prediction and homolog-based methods, we identified and classified 1.18 Gb of repetitive sequences, taking up 72.51% of the assembled genome, mainly including 7.99% DNA elements, 34.08% long interspersed elements (LINE), and 16.35% unknown sequences (Tables 4 & 5). The repeat-content in the *M. bilineatum* genome is similar to the Spanish slug *A. vulgaris* (75.09%), and is higher than other studied gastropod species^28,33^. These results further validate the accuracy of our genome assembly.

**Table 4:** Repetitive sequences statistics for the *M. bilineatum* genome.

	Repeat size (bp)	Percentage of genome (%)
Trf	317,268,000	19.46
Repeatmasker	222,324,360	13.64
Proteinmask	246,028,476	15.09
*De novo*	822,410,502	50.44
Total	1,182,234,746	72.51

**Table 5:** Transposable elements statistics for the *M. bilineatum* genome.

	RepBase TEs		TE Proteins		*De novo*		Combined TEs	
	Length (bp)	Percentage of genome (%)	Length (bp)	Percentage of genome (%)	Length (bp)	Percentage of genome (%)	Length (bp)	Percentage of genome (%)
DNA	60,821,580	3.73	15,758,629	0.97	81,586,513	5.00	130,206,628	7.99
LINE	150,309,599	9.22	230,097,164	14.11	472,372,895	28.97	555,630,054	34.08
SINE	1,308,004	0.08	0	0.00	8,594,228	0.53	9,807,121	0.60
LTR	15,236,929	0.93	215,940	0.01	7,872,522	0.48	22,997,099	1.41
Satellite	5,671,878	0.35	0	0.00	2,144,445	0.13	7,760,239	0.48
Simple_repeat	0	0.00	0	0.00	386,577	0.02	386,577	0.02
Other	11,377	0.00	0	0.00	0	0.00	11,377	0.00
Unknown	1,417,810	0.09	0	0.00	265,281,286	16.27	266,631,547	16.35
Total	222,324,360	13.64	246,028,476	15.09	822,410,502	50.44	957,005,244	58.69

### Identification and annotation of protein-coding genes

**Fig. 3:**
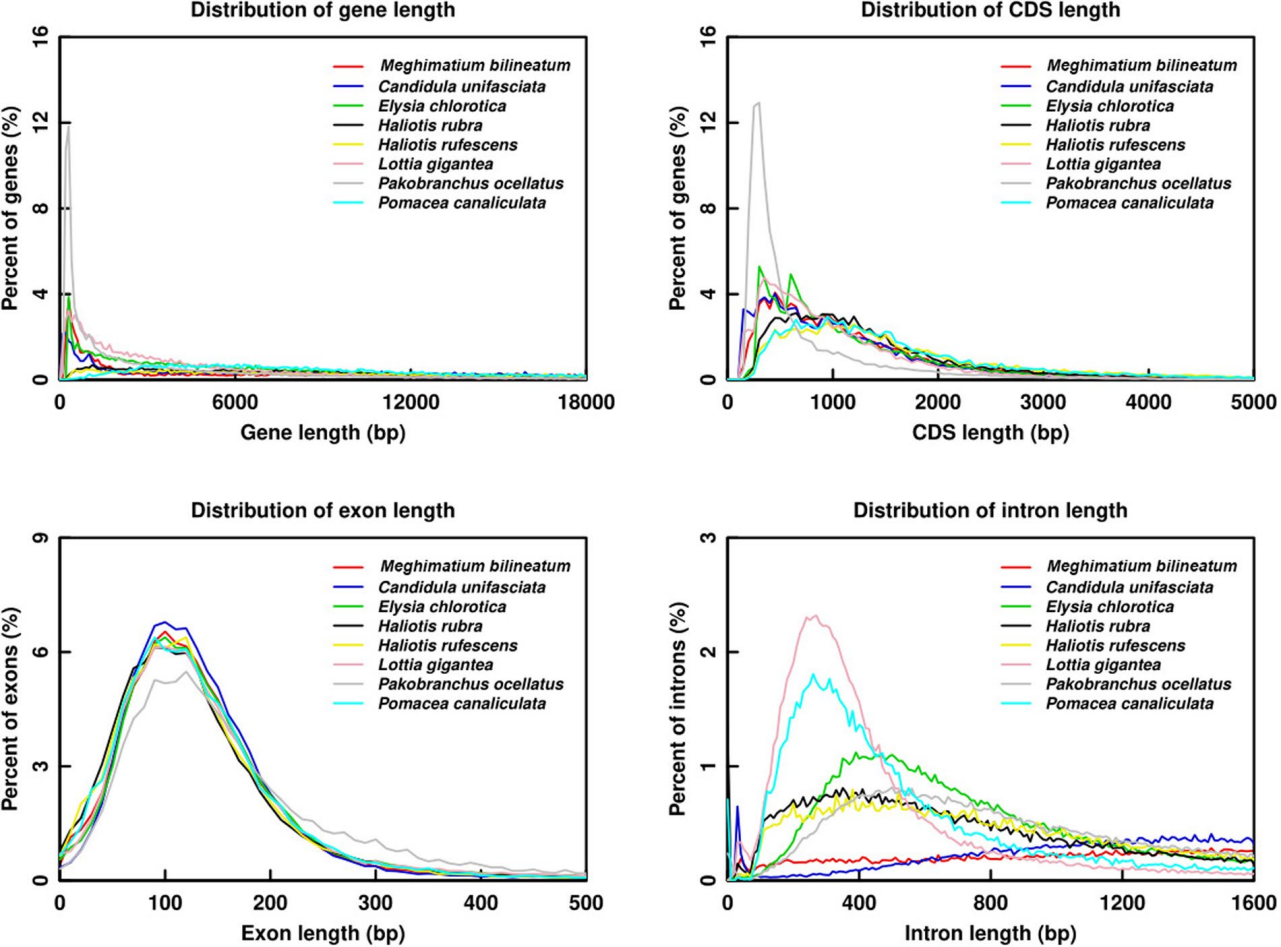
Comparison of protein-coding genes annotation quality. Eight species (*M. bilineatum, Haliotis rufescens, Pakobranchus ocellatus, Lottia gigantea, Candidulaunifasciata, Elysia chlorotica, Haliotis rubra*, and *Pomacea canaliculate*) were examined to compare the lengths of the gene, CDS, exon, and intron.

First, we used repeat-masked genome sequences to perform *ab initio* gene prediction, and then used AUGUSTUS (v3.3.2), Genscan (v1.0) and GlimmerHMM (v3.0.4) software to detect the protein-coding genes^34–36^. Second, to conduct homology-based prediction, protein sequences from *Candidula unifasciata* (GCA_905116865.2), *Elysia chlorotica* (GCA_003991915.1), *Haliotis rubra* (GCA_003918875.1), *Haliotis rufescens* (GCA_023055435.1), *Lottia gigantea* (GCA_000327385.1), *Pakobranchus ocellatus* (GCA_019648995.1), and *Pomacea canaliculate* (GCA_003073045.1) were compared with the *M. bilineatum* genome utilizing TBLASTN (v2.2.29) (e-value ≤ 1e^-5^) to determine candidate regions, and further used GenWise (v2.4.1) software to accurately map the screened proteins to the *M. bilineatum* genome to obtain splice sites^37^. Third, to perform transcriptome sequencing-based prediction, the RNA-seq reads from Illumina were mapped to the *M. bilineatum* genome by using the TopHat (v2.1.1) software following default arguments, and the transcripts were assembled using Cufflinks (v2.2.1) software with the “-e 100 -C” parameter^38,39^, and the protein-coding genes were determined using the PASA (v2.3.2)^40^. Fourth, using the MAKER2 (v2.31.10) and HiFAP software following default parameters, we combined the three predictions to construct a complete and nonredundant reference gene database^41^. Finally, in the *M. bilineatum* genome, 18631 identified protein-coding genes were found. The length of the average gene, including CDS, exon, and intron, is presented in Table 6. These predicted gene structures were also compared with the seven other homologous species (Fig. 3).

**Table 6:** Statistics on transposable elements in the *M. bilineatum* genome.

	Gene set	Protein coding gene number	Average gene length (bp)	Average CDS length (bp)	Average exon per gene	Average exon length (bp)	Average intron length (bp)
*de novo*	Genscan	28,980	29,601	1,520	5.74	264.66	5,918
	AUGUSTUS	22,383	11,083	1,024	4.25	240.82	3,094
Homolog	*Haliotis rufescens*	41,610	18,636	774.74	3.91	198.29	6,144
	*Pakobranchusocellatus*	70,916	10,866	580.26	2.73	212.23	5,931
	*Lottia gigantea*	40,785	11,419	613.66	3.08	199.44	5,203
	*Candidulaunifasciata*	55,090	12,686	756.41	3.72	203.53	4,392
	*Elysia chlorotica*	81,057	6,905	562.17	2.26	248.88	5,039
	*Haliotis rubra*	41,094	15,205	751.1	3.59	208.94	5,570
	*Pomacea canaliculata*	35,097	18,984	742.64	4.19	177.36	5,723
RNAseq	Transdecoder	486	19,342	807.33	5.72	226.07	3,824
BUSCO		5,814	31,916	1,746	11.73	148.91	2,813
MAKER		32,859	9,954	643.25	3.73	181.39	3,397
HiFAP		18,816	20,566	1,313	6.85	196.24	3,287

We annotated these protein-coding genes functions through the alignment of gene sequences to the InterPro, GO, KEGG, SwissProt, TrEMBL, TF, Pfam, NR, and KOG database by using BLAST + (2.11.0) software (e-value ≤ 1e^-5^)^42–47^. In addition, based on InterPro database and Pfam database, the conserved protein domain and motif associated with the function annotated was determined using the InterProScan tool (v5.61-93.0) with the “-seqtype p -formats TSV -goterms -pathways -dp” parameter^48^. Ultimately, a total of 15569 genes (83.57%) were successfully annotated (Table 7).

**Table 7:** Putative protein-coding gene functional annotations of the *M. bilineatum* genome.

Database	Annotated number of putative genes	Percent (%)
InterPro	13,162	69.95
GO	9,689	51.49
KEGG_ALL	13,858	73.65
KEGG_KO	9,270	49.27
Swissprot	11,254	59.81
TrEMBL	15,338	81.52
TF	1,426	7.58
Pfam	12,474	66.29
NR	15,258	81.09
KOG	10,894	57.9
All annotated	15726	83.58
Predicted genes	18816	

### Identification of non-coding genes

The tRNA, rRNA, miRNA, and snRNA non-coding RNAs are not translated into proteins. In the annotation process of non-coding RNAs, tRNAscan-SE (v1.3.1) software following the default parameters was used to find the tRNA sequences in the assembled genome according to the structural characteristics of tRNA^49^. BLASTN was applied to identify rRNA genes in the assembled genome according to the highly conserved characteristics of rRNA. In addition, according to the covariance model of Rfam database (v14.8), we used the INFERNAL program with default arguments to predict the miRNA and snRNA sequences^50^. Finally, 1424 rRNAs, 941 tRNAs, 588 snRNAs, and 49 miRNAs were annotated (Table 8).

**Table 8:** Statistics of the noncoding RNA in the *M. bilineatum* genome.

Type		Copy	Average length(bp)	Total length(bp)	% of genome
miRNA		49	83	4,074	0.00025
tRNA		941	75	70,126	0.004301
rRNA	rRNA	1,424	608	866,300	0.053131
	18 S	693	1,105	765,478	0.046948
	28 S	225	145	32,641	0.002002
	5.8 S	241	154	37,030	0.002271
	5 S	265	118	31,151	0.001911
snRNA	snRNA	588	150	87,935	0.005393
	CD-box	292	154	45,041	0.002762
	HACA-box	31	162	5,011	0.000307
	splicing	258	143	36,974	0.002268
	scaRNA	7	130	909	0.000056

### Comparative genomic analysis

**Fig. 4:**
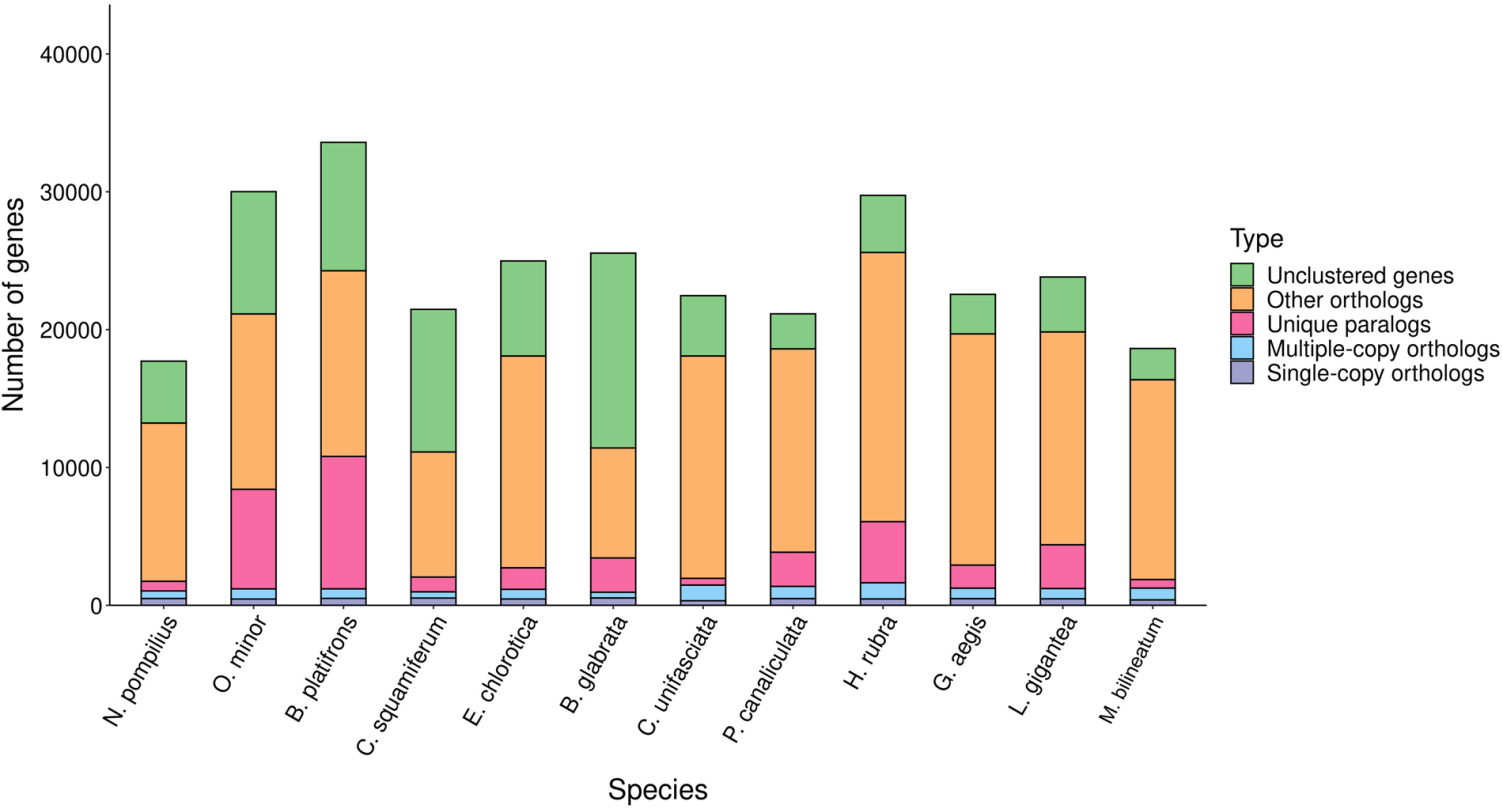
Distribution of genes in different species.

**Fig. 5:**
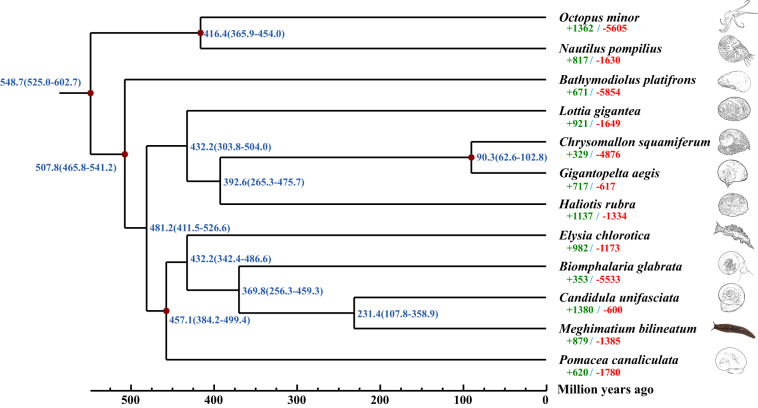
Phylogenetic analysis of *M. bilineatum* and 11 other mollusks. The green and red numbers on each branch represent the number of significantly expanded and contracted gene families, respectively. The blue numbers on each branch represent the divergence time (MYA) of these 12 mollusks.

The single-copy ortholog genes of *M. bilineatum* and 11 other molluscan species (Table S3), including *Nautilus pompilius*, *Octopus minor*, *Bathymodiolus platifrons*, *Chrysomallon squamiferum*, *Elysia chlorotica*, *Biomphalaria glabrata*, *Candidula unifasciata*, *Pomacea canaliculate*, *Haliotis rubra*, *Gigantopelta aegis* and *Lottia gigantea*, were determined using the “-l 1.5” parameter of hcluster_sq software from OrthoMCL (v2.0.9) to validate the phylogenetic relationships among the 12 molluscan species^51^. A total of 29157 gene families were determined, including 671 common orthologous gene families and 135 single-copy gene families, in the 12 molluscan species (Fig. 4; Table S4). The MAFFT (v7.487) software with default parameters was used to compare the single-copy genes^52^. All conserved sequences in the single-copy genes were extracted using Gblock (v0.91b) software with the “-t = c” parameter^53^. Subsequently, the ML phylogenetic tree was constructed using the “-f a -N 100 -m GTRGAMMA” parameter of RAxML (v8.2.12)^54^, with *N. pompilius* and *O. minor* as the outgroup. Moreover, the divergence time of the 12 mollusks were estimated using the MCMCtree (v4.4) program in software PAML (v4.9) with “clock = 3; model = 0” parameter according to the calibration times of *N. pompilius*-*B. platifrons* (619.1–527.6 MYA), *B. platifrons*-*P. canaliculata* (541.7–463.4 MYA), *N. pompilius*-*O. minor* (452.6–364.2 MYA), *B. glabrata*-*P. canaliculata* (496.0–310.0 MYA) and *G. aegis*-*C. squamiferum* (100.0–42.4 MYA) from the Timetree database^55^. The evolutionary tree showed that *M. bilineatum* and *C. unifasciata* were clustered together, and diverged ~231.4 MYA (Fig. 5). We also identified the expanded genes and contracted gene families in the 12 mollusks using CAFE (v5.0.0) with the “-p 0.05 -t 4 -r 10000” parameter^56^. The result showed that there were 879 expanded gene families and 1385 contracted gene families in the *M. bilineatum* (Fig. 5).

## Data Records

All sequencing data from three sequencing platforms have been uploaded to the NCBI SRA database (transcriptomic sequencing data: SRR25867028^57^, genomic Illumina sequencing data: SRR25903989^58^, genomic PacBio sequencing data: SRR25919044^59^ and SRR25919043^60^, Hi-C sequencing data: SRR25919155^61^ and SRR25919154^62^). The final chromosome-level assembled genome file has been uploaded to the GenBank database under the accession JAXGFX000000000^63^. Genome annotation files (including repeat-content annotation, gene structure annotation, gene functional annotation and non-coding genes annotation) have been uploaded to the Figshare database^64^.

## Technical Validation

### Evaluating quality of the DNA and RNA

Prior to the genome sequencing, we used the NanoDrop 2000 Spectrophotometer (Thermo Fisher Scientific, San Jose, CA, USA) and Qubit 3.0 Fluorometer (Thermo Fisher Scientific, San Jose, CA, USA) to determine the quality (OD260/280 and OD260/230) and concentration of the DNA and RNA samples to ensure the accuracy of sequencing data. We also used the agarose gel electrophoresis and Agilent 2100 Bioanalyzer (Agilent Technologies, Palo Alto, California, USA) to determine the integrity of the DNA and RNA samples.

### Evaluating quality of the genome assembly

To evaluate the sequence consistency and assembly quality, the BWA (v0.7.17-r1188) and Minimap2 (v2.24_x64-linux) software were used to map the short reads from Illumina and HiFi reads from PacBio to the assembled genome, respectively^65,66^. After these processes, 99.35% of the short reads from Illumina and 99.62% of the HiFi reads from PacBio were aligned, covering 99.81% and 99.99% of the assembled genome, respectively (Table S5 & S6). Moreover, BUSCO (v5.4.3) analysis was conducted to evaluate the assembly quality based on the mollusca_odb10 database^67^. A total of 91.70% of the 5295 single-copy orthologs in the assembled genome were determined as complete, including 4015 single-copy (75.80%) and 842 duplicated (15.90%), 0.89% and 7.46% of the total single-copy orthologs were fragmented and missing, respectively (Table 9).

**Table 9:** Results of BUSCO analysis of the *M. bilineatum* genome.

	Assembly		Annotation	
Type	Proteins	Percentage (%)	Proteins	Percentage (%)
Complete BUSCOs (C)	4,857	91.70	4,851	91.60
Single-copy BUSCOs (S)	4,015	75.80	3,912	73.90
Duplicated BUSCOs (D)	842	15.90	939	17.70
Fragmented BUSCOs (F)	44	0.80	70	1.30
Missing BUSCOs (M)	394	7.50	374	7.10
Total BUSCOs	5,295	100.00	5,295	100.00

### Evaluating quality of the genome annotation

BUSCO (v5.4.3) analysis was conducted to evaluate the genome annotation quality based on the mollusca_odb10 database^67^. A total of 91.60% of the 5295 single-copy ortholog genes in the assembled genome were determined as complete, including 3912 single-copy genes (73.90%) and 939 duplicated genes (17.70%), 1.30% and 7.10% of the total genes were fragmented and missing, respectively (Table 9).

### Supplementary information

Supplementary information of Chromosomal-scale genome assembly and annotation of the land slug (*Meghimatium bilineatum*)

## Data Availability

No specific code was used in this study. The standard bioinformatic tools were used for data analysis. Furthermore, the parameter setting of the bioinformatics tools was performed in accordance with the manual and protocols and described in the Methods Section.
