# Supplementary material for: Chromosomal-scale genome assembly and annotation of the land slug (Meghimatium bilineatum)
Source: Sci Data. 2024 Jan 5;11:35. doi: 10.1038/s41597-023-02893-7 (PMC10770140; doi:10.1038/s41597-023-02893-7)
Supplement: Supplementary file 1 — Supplementary information of Chromosomal-scale genome assembly and annotation of the land slug (Meghimatium bilineatum) [file 41597_2023_2893_MOESM1_ESM.docx]

**Supplementary information of Chromosomal-scale genome assembly and annotation of the** **land slug (*Meghimatium bilineatum*)**

| Table S1 Statistics of alignment results of clean paired-end reads. | Page 2 |
| --- | --- |
| Table S2 Statistics of valid paired-end reads. | Page 2 |
| Table S3 Species information used in the comparative genomic analysis. | Page 3 |
| Table S4 Statistics of the gene families of the twelve molluscan species. | Page 4 |
| Table S5 The alignment of the Illumina reads to the slug genome assembly | Page 4 |
| Table S6 The alignment of the Pacbio subreads to the slug genome assembly | Page 4 |

**Table S1 Statistics of alignment results of clean paired-end reads.**

|  | Read 1 | | Read 2 | |
| --- | --- | --- | --- | --- |
|  | Number | Percentage (%) | Number | Percentage (%) |
| Unique alignments | 298,250,881 | 63.1 | 276,428,508 | 58.49 |
| Multiple alignments | 107,779,925 | 22.8 | 116,217,307 | 24.59 |
| Too short to align | 29,366,027 | 6.21 | 41,899,949 | 8.87 |
| Filed to align | 37,247,310 | 7.88 | 38,098,379 | 8.06 |
| Paired | 185,356,467 | 39.22 | 185,356,467 | 39.22 |

**Table S2 Statistics of valid paired-end reads.**

| Species | Di-Tag Count | Percent in Paired (%) | Percent in Total Reads(%) |
| --- | --- | --- | --- |
| Same Circularised | 418,466 | 0.23 | 0.09 |
| Same Fragment Dangling Ends | 2,578,685 | 1.39 | 0.55 |
| Same Fragment Internal | 14,800,398 | 7.98 | 3.13 |
| Re-ligation | 1,249,436 | 0.67 | 0.26 |
| Contiguous Sequence | 0 | 0 | 0 |
| Wrong Size | 0 | 0 | 0 |
| Invalid Pairs | 19,046,985 | 10.28 | 4.03 |
| Valid Pairs | 166,309,482 | 89.72 | 35.19 |
| Valid Pairs (de-duplication) | 163,147,965 | 88.02 | 34.52 |

**Table S3** **Species information used in the comparative genomic analysis.**

| Species | Family | Habitat type | Data source |
| --- | --- | --- | --- |
| *Nautilus pompilius* | Nautilidae | Marine | <http://mgbase.qnlm.ac/downloadData/download?path=Nautilus_pompilius.cds.fa> |
| *Octopus minor* | Octopodidae | Marine | <http://mgbase.qnlm.ac/downloadData/download?path=Octopus_minor.cds.fa> |
| *Bathymodiolus platifrons* | Mytilidae | Marine | <http://mgbase.qnlm.ac/downloadData/download?path=Bathymodiolus_platifrons.cds.fa> |
| *Chrysomallon squamiferum* | Peltospiridae | Marine | <http://mgbase.qnlm.ac/downloadData/download?path=Chrysomallon_squamiferum_v2.0.cds.fa> |
| *Elysia chlorotica* | Plakobranchidae | Marine | <http://mgbase.qnlm.ac/downloadData/download?path=Elysia_chlorotica.cds.fa> |
| *Biomphalaria glabrata* | Planorbidae | Terrestrial | <http://mgbase.qnlm.ac/downloadData/download?path=Biomphalaria_glabrata.cds.fa> |
| *Candidula unifasciata* | Geomitridae | Marine | <https://www.ncbi.nlm.nih.gov/datasets/taxonomy/100452/> |
| *Pomacea canaliculate* | Ampullariidae | Terrestrial | <https://www.ncbi.nlm.nih.gov/datasets/taxonomy/400727/> |
| *Haliotis rubra* | Haliotidae | Marine | <https://www.ncbi.nlm.nih.gov/datasets/taxonomy/36100/> |
| *Gigantopelta aegis* | Peltospiridae | Marine | <https://www.ncbi.nlm.nih.gov/datasets/taxonomy/1735272/> |
| *Lottia gigantea* | Lottiidae | Marine | <https://www.ncbi.nlm.nih.gov/datasets/taxonomy/225164/> |

**Table S4 Statistics of the gene families of the twelve molluscan species.**

| Species | Genes number | Genes in families | Unclustered genes | Family number | Unique families | Common families | Single copy | Average_genes_per_family |
| --- | --- | --- | --- | --- | --- | --- | --- | --- |
| *Nautilus pompilius* | 17,710 | 13,221 | 4,489 | 10,756 | 240 | 671 | 135 | 1.229 |
| *Octopus minor* | 30,010 | 21,138 | 8,872 | 8,031 | 1,507 | 671 | 135 | 2.632 |
| *Bathymodiolus platifrons* | 33,584 | 24,273 | 9,311 | 10,490 | 2,215 | 671 | 135 | 2.314 |
| *Chrysomallon squamiferum* | 21,469 | 11,125 | 10,344 | 8,731 | 389 | 671 | 135 | 1.274 |
| *Elysia chlorotica* | 24,980 | 18,088 | 6,892 | 13,621 | 461 | 671 | 135 | 1.328 |
| *Biomphalaria glabrata* | 25,550 | 11,414 | 14,136 | 8,059 | 883 | 671 | 135 | 1.416 |
| *Candidula unifasciata* | 22,464 | 18,090 | 4,374 | 13,038 | 170 | 671 | 135 | 1.387 |
| *Pomacea canaliculate* | 21,144 | 18,606 | 2,538 | 12,034 | 480 | 671 | 135 | 1.546 |
| *Haliotis rubra* | 29,738 | 25,596 | 4,142 | 14,403 | 1,103 | 671 | 135 | 1.777 |
| *Gigantopelta aegis* | 22,556 | 19,691 | 2,865 | 13,682 | 447 | 671 | 135 | 1.439 |
| *Lottia gigantea* | 23,818 | 19,832 | 3,986 | 13,089 | 576 | 671 | 135 | 1.515 |
| *M. bilineatum* | 18,631 | 16,368 | 2,263 | 11,471 | 150 | 671 | 135 | 1.427 |

**Table S5 The alignment of the Illumina reads to the *M. bilineatum* genome assembly.**

| Mapping  Rate (%) | Paired mapping rate (%) | Average  sequencing  depth | Coverage (%) | Coverage  at least 4X (%) | Coverage  at least 10X (%) | Coverage  at least 20X (%) |
| --- | --- | --- | --- | --- | --- | --- |
| 99.35 | 96.40 | 159.12 | 99.81 | 99.60 | 99.33 | 98.95 |

**Table S6 The alignment of the Pacbio subreads to the *M. bilineatum* genome assembly.**

| Mapping  Rate (%) | Paired mapping rate (%) | Average  sequencing  depth | Coverage (%) | Coverage  at least 4X (%) | Coverage  at least 10X (%) | Coverage  at least 20X (%) |
| --- | --- | --- | --- | --- | --- | --- |
| 99.62 | 41.82 | 99.99 | 99.04 | 94.73 | 82.78 | 99.62 |
